# Cardiotrophin-1 in Asymptomatic Hypertensive Patients With Mild Diastolic Dysfunction: Potential Prognostic Value in Early Stages of Hypertensive Heart Disease

**DOI:** 10.7759/cureus.46516

**Published:** 2023-10-05

**Authors:** Ioannis Vlahodimitris, Dimos Karangelis, Maria Moschaki, Ioannis Moyssakis, Konstantinos C Christodoulou, Despoina N Perrea, Iordanis Mourouzis, Dimitrios Papadogiannis

**Affiliations:** 1 Cardiology, Laiko General Hospital of Athens, Athens, GRC; 2 Cardiothoracic Surgery, Democritus University of Thrace, Alexandroupolis, GRC; 3 Anesthesia, Evangelismos Hospital of Athens, Athens, GRC; 4 Laboratory of Experimental Surgery and Surgical Research, National and Kapodistrian University of Athens School of Medicine, Athens, GRC; 5 Pharmacology, National and Kapodistrian University of Athens, Athens, GRC; 6 First Department of Propaedeutic Medicine, Laiko General Hospital of Athens, National and Kapodistrian University of Athens, Athens, GRC

**Keywords:** heart failure, bnp, hypertension, diastolic dysfunction, cardiotrophin-1

## Abstract

Background: Regardless of the advancements in modern technology and treatment options, heart failure (HF) exhibits impervious mortality and morbidity rates. Arterial hypertension poses one of the greatest risks for developing HF, yet the exact pathophysiological path and changes that lead from isolated hypertension to HF are still unclear. Cardiotrophin-1 (CT-1) serves as a promising prognostic biomarker for the onset of HF in hypertensive patients. The aim of this study was to investigate whether CT-1 levels are elevated in a selected group of asymptomatic hypertensive patients.

Methods: In a selected cohort of 40 asymptomatic patients with early diastolic dysfunction (grade I), without any signs of increased filling pressures in the left ventricle, as well as 20 healthy individuals, the levels of CT-1 brain natriuretic peptide (BNP) along with various echocardiographic parameters were evaluated.

Results: The mean age of the hypertensive patients was 56 ± 5 years and 52± 3.5 years for the normotensive controls. The hypertensive group exhibited higher levels of CT-1, which was not affected by left ventricular hypertrophy. Notably, in patients with normal E/E′ < 8 (n = 30), CT-1 levels were 1165 ± 471 pg/ml compared to 2069 ± 576 pg/ml in patients with marginal E/E′ > 8 and <14 (n = 10), p = 0.001.

Conclusions: Our study demonstrated elevated CT-1 levels in a cohort of asymptomatic hypertensive patients, exhibiting mild diastolic dysfunction. These findings are suggestive of the potentially prognostic value of this particular biomarker in the early stages of hypertensive heart disease.

## Introduction

Heart failure (HF) is a major health problem affecting millions of patients worldwide [1,2]. Regardless of the advancements in modern technology and treatment options, it exhibits impervious mortality and morbidity rates [2]. Through the induction of left ventricular hypertrophy (LVH) and coronary atherosclerosis, arterial hypertension poses one of the greatest risks for developing HF [3,4]. However, the severity of hypertension does not predict the risk of developing hypertensive heart disease, leading to functional and structural changes and eventually in HF [5]. Thus, the factors that determine the progress from isolated hypertension toward hypertensive heart disease and HF, in some patients, are not fully understood [6]. Furthermore, biomarkers predicting this adverse prognosis during the early stages of the disease would be valuable in clinical practice.

As a response to hypoxia, neurohumoral factors (norepinephrine, angiotensin II, aldosterone) or even mechanical stretch, cardiomyocytes, and myofibroblasts produce cardiotrophin-1 (CT-1), a cytokine of the IL-6 suprafamily, with a molecular weight of 22.5 kDa, which is activated through the glycoprotein 130 (gp130)/leukemia inhibitory factor receptor heterodimer [7]. In addition to serving as a response factor to stress and facilitating short-term survival via antiapoptotic mechanisms, CT-1 has been shown to exert a pathophysiological role in the development of HF [8]. Experimental studies reported that prolonged exposure to CT-1 impairs the contractile function of cardiomyocytes [9], probably via disrupted calcium handling [10]. In clinical studies, elevated levels of CT-1 have been associated with LVH and eccentric hypertrophy [7,8,11]. Thus CT-1 has been shown to produce in-line accumulation of sarcomeres in cardiac cells [11,12], without concomitant increase in sarcomere width [13]. Altogether, CT-1 serves as a promising prognostic biomarker for the onset of HF in hypertensive patients.

In the present study, the authors sought to investigate whether CT-1 levels are elevated in a selected group of asymptomatic hypertensive patients with early diastolic dysfunction (grade I) but without any signs of increased filling pressures in the left ventricle (LV). Furthermore, factors contributing to this increase will be also investigated.

## Materials and methods

Study population

Informed consent was obtained from all patients. The study was held in the Cardiology Clinic of Laiko General Hospital of Athens and received approval by the Ethics Committee (#ES255, 27/04/2009). We identified all hypertensive patients who were scheduled for a routine medical work-up between 2009 and 2011. A positive history of chronic antihypertensive medication and the detection of at least three times systolic blood pressure and diastolic blood pressure values over 139 mmHg and/or 89 mmHg, respectively, were used to identify hypertension. Only asymptomatic hypertensive patients under 65 years of age, with initial diastolic dysfunction grade I (prolonged relaxation), were included in the study. All patients were submitted to clinical and laboratory evaluations to exclude secondary hypertension.

Patients with symptoms or history of HF, hypertrophic cardiomyopathy, coronary artery disease, aortic stenosis, cerebrovascular disease, peripheral artery disease, and segmental abnormalities of wall motion in echocardiography were excluded, as were patients with left ventricular ejection fraction (LVEF) less than 50%. Regarding LVH, the definition was based on septal wall thickness, and the cut-off value was set to 0.9 cm for females and 1.0 cm for males according to the most recent guidelines. Any clinical variables that could affect the outcome of the present study, such as cancer, surgery autoimmune disease, or comorbidities, were considered confounding variables and therefore were excluded.

Population Comparison Cohort

A control cohort was sampled from healthy individuals who were scheduled for a routine physical examination. The systolic blood pressure range for the control cohort was set at 120-139 mmHg, and the diastolic was set at 80-89 mmHg.

Echocardiography

All of the study participants underwent echocardiographic evaluation with measured mitral inflow pulse wave Doppler parameters, including peak velocity of early diastolic filling (E), late fill­ing (A), and deceleration time (DT) of the E-wave velocity and isovolumetric relaxation time (IVRT). The aforementioned parame­ters were obtained, during diastole, employing the apical four-chamber view and placing a sample volume between the mitral leaf­let tips. We also measured early dia­stolic mitral annular velocity (Em) with tissue Doppler parameter, which was from the apical four-chamber view with sample volume placed 1 cm within the septal and lateral insertions of the mitral leaflets (E′ s - septal, E′ l - lateral). Using Doppler, we measured the Tei index. For left ventricular mass, we calculated the intraventricular septal (IVS) and posterior wall of the left ventricle (PWLV), using the Devereux method [14].

Left atrial (LA) volume was calculated with the area-length formula:

$\left (\frac{8}{3} \right )\pi\left [\frac{(A\ \times\ A2)}{L} \right ]$

where A1 is the area of LA in the four-chamber view (4C). In this specific examination, we accept A1 = A2, where A2 is the area of the LA in the two-chamber view (2C); L is the LA long-axis length determined as the distance of the perpendicular line measured from the middle of the plane of the mitral annulus to the superior aspect of the LA [15]. Diastolic dysfunction grade I was calculated by the mitral inflow as a ratio, E/A</0.8 plus E </50cm/sec [16].

All patients were evaluated for somatometric data, including exact height, weight, and age. Body surface area (BSA) was calculated using the following equation:

$\sqrt{\frac{(Weight\ in\ kg\ \times\ Height\ in\ cm)}{3600}}$

LA index was calculated using the following equation:

$\frac{LA\ volume}{BSA}$

Biomarker evaluation

Peripheral blood was drawn from all subjects, using an aseptic technique. The concentration of CT-1 (pg/ml) was measured by enzyme-linked immunosorbent assay (ELISA). With an intra-assay coefficient of variation of less than 10%, all samples were measured concurrently. The lower level of detection was 5.9 pg/mL. Brain natriuretic peptide (BNP, pg/ml) was determined in plasma samples by ELISA as instructed by the manufacturer (SEA541Hu, USCN Life Science Inc., Houston, Texas). The intra-assay coefficient of variation was less than 7%.

Study endpoints and sample size calculation

The primary endpoint of the study was an increase of ≥25% in CT-1 in hypertensive patients compared to normotensive controls. The mean ± standard deviation (SD) of CT-1 levels in the control group was roughly estimated as 1000 ± 295 pg/ml. For every eligible member of the general population cohort, two hypertensive patients were selected. With these assumptions and an allocation ratio of 2:1 in favor of the hypertensive group, a sample size of 60 patients (that indicates 40 subjects for hypertensive and 20 subjects for control group) was estimated to detect a mean increase of 25% in CT-1 between the two study groups with 86% power. The criterion for significance (alpha) was set at 0.05, and the test was two-tailed.

Statistical analysis

Values are reported as means ± SD. To assess the differences between normotensive and hypertensive patients as well as between hypertensive individuals with or without LVH, the student’s t-test for unpaired data was used. If normality was not confirmed, a Mann-Whitney U-test was employed. Binary data were evaluated using the x^2^ test. Once normality was confirmed, univariate regression analysis was utilized to quantify the relationships using the Pearson correlation coefficient. After the logarithmic adjustment, variables with nonparametric distributions were examined. Statistical analysis was carried out using SPSS v17.0 (IBM Corp., Armonk, NY). Statistical significance was defined as two-sided (p < 0.05).

## Results

Patient baseline characteristics

We identified 40 consecutive hypertensive patients, while the comparison cohort consisted of 20 individuals. The mean age was 56 ± 5 years for hypertensive patients and 52 ± 3.5 years for the normotensive controls (p = 0.001). Female patients represented 42.5% (17/40) in the hypertensive group and 33.3% (7/21) in the control group (p = 0.48). Hypertensive patients exhibited higher BMI as compared to normotensive patients (28.7 ± 3.7 versus 26.3 ± 3.4, p = 0.013). LVEF and left ventricular end-diastolic diameter (LVEDD) were normal for both groups (>50% and <55 mm, respectively). Patients at the time of examination had been on antihypertensive treatment as follows: 45% of patients received angiotensin-converting enzyme inhibitors (ACEIs) or angiotensin receptor blockers as monotherapy, 15% received β-blockers, 15% received calcium channel blockers, and 25% received a combination of angiotensin receptor blockers with calcium channel blockers. In the control cohort, all members had multiple normal blood pressure, echocardiography, and laboratory findings, with no prior history of cardiac disease.

Echocardiographic parameters

Initially, we sought to investigate the DT, isovolumic relaxation time (IVRT), intraventricular septal (IVS), PWLV, Tei index, and mitral wave velocities (E, A), between controls and patients, as these indices differentiate these two groups in view of diastolic dysfunction. Next, we evaluated mitral annular velocities (E′s - septal, E′ l - lateral) and E/A ratio in order to establish grade I diastolic dysfunction in patients.

Patients differed significantly from controls in all echocardiographic indices of diastolic function as shown in Table 1. In fact, patients had significantly higher DT, IVRT, IVS, and PWLV than controls, while E′s, E′l, E/A, and Tei index were all significantly lower than controls. The ratio of transmitral Doppler early filling velocity to tissue Doppler early diastolic mitral annular velocity (E/mean E′ [septal + lateral]) was only slightly increased in hypertensives versus controls, while no patient had E/E′ greater than 14 indicating that diastolic filling pressures were within normal range in all patients. In addition, the LA volume was significantly higher in the patient group compared to the controls.

**Table 1:** Echocardiographic parameters Values are reported as means ± SD. DT: Deceleration time; IVRT: Isovolumetric relaxation time; IVS: Intraventricular septal; PWLV: Posterior wall of the left ventricle; TDI: Tissue Doppler imaging; E/A: Early diastolic filling/late fill­ing; LA: Left atrial.

	Patients (N = 40)	Controls (N = 21)	p-value
DT (ms)	193.0 ± 26	156 ± 10	<0.001
IVRT (ms)	89 ± 9.5	75 ± 5.9	<0.001
IVS (cm)	1.07 ± 0.10	0.86 ± 0.10	<0.001
PWLV (cm)	1.07 ± 0.1	0.90 ± 0.09	<0.001
TEI index	0.43 ± 0.04	0.37 ± 0.02	<0.001
Septal TDI E′ velocity (cm/s)	8.5 ± 2.2	10.6 ± 1.3	<0.001
Lateral TDI E′ velocity (cm/s)	9.4 ± 2.0	13.5 ± 2.1	<0.001
E/mean TDI E′ velocity	7.1 ± 1.9	6.2 ± 1.3	0.06
E/A	0.8 ± 0.09	1.19 ± 0.11	<0.001
LA volume (ml)	30.7 ± 4.5	26.5 ± 4.1	0.001

Biomarkers

Peripheral blood levels of CT-1 and BNP were assessed. As shown in Table 2, BNP levels were slightly higher in hypertensives as compared to controls; however, subjects in both groups had BNP levels within a normal range (below 100 pg/ml) indicating that no patients with HF were included in this study.

**Table 2:** Biomarker levels Values are reported as means ± SD. BNP: Brain natriuretic peptide.

	Patients (N = 40)	Controls (N = 21)	p-value
Cardiotrophin-1 (pg/ml)	1371 ± 662	1124 ± 246	0.04
BNP (pg/ml)	48 ± 28	34 ± 17	0.02

**Figure 1:**
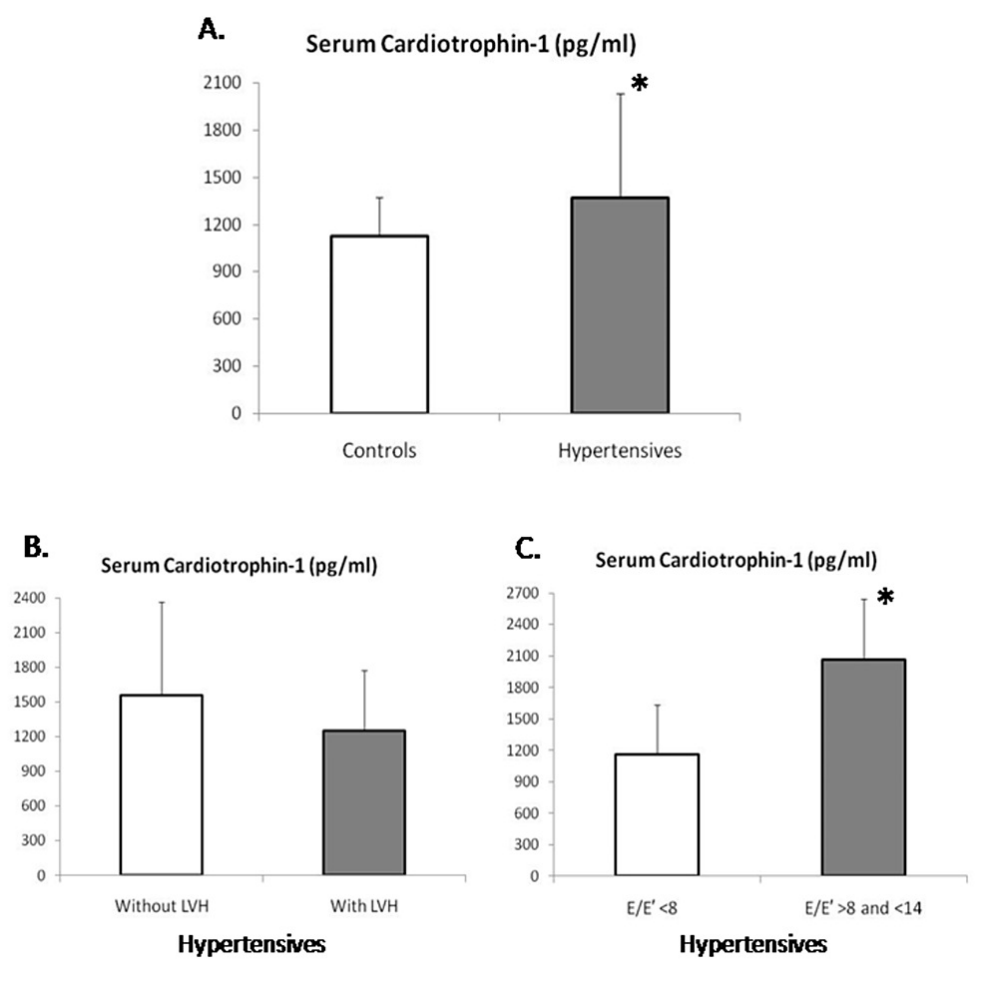
Cardiotrophin-1 levels across arm groups (A) Cardiotrophin-1 levels in controls and hypertensive patients with mild diastolic dysfunction. (B) Cardiotrophin-1 levels in hypertensive patients with and without left ventricular hypertrophy (LVH). (C) Cardiotrophin-1 levels in hypertensive patients with E/E′ < 8 (normal diastolic filling pressure) and E/E′ > 8 and <14 (borderline diastolic filling pressure). * p < 0.05.

Compared to normotensive controls, the levels of CT-1 were significantly elevated in hypertensive patients (Table 2). In order to provide further insights into the possible causes of this increased production of CT-1, we examined correlations of CT-1 levels with other echocardiographic parameters. As CT-1 is known to correlate with the development of LV hypertrophy, we examined the levels of CT-1 in hypertensive patients with and without hypertrophy. In fact, CT-1 levels were 1555 ± 808 pg/ml in patients without hypertrophy (n = 16) versus 1248 ± 528 pg/ml in patients with hypertrophy (n = 24), p = NS. No other significant correlation was found between CT-1 and other echocardiographic parameters of diastolic function. Since the index (E/mean value of TDI E′) is an important indicator of diastolic filling pressures, we examined whether CT-1 levels were different in patients with normal E/E′ < 8 and marginal E/E′ > 8 and <14. Notably, CT-1 levels were 1165 ± 471 pg/ml in patients with normal E/E′ < 8 (n = 30) versus 2069 ± 576 pg/ml in patients with marginal E/E′ > 8 and <14 (n = 10), p = 0.001 (Figure 1).

## Discussion

The present study evaluated the levels of CT-1 in a group of asymptomatic hypertensive patients that were examined in order to characterize left ventricular diastolic function by methods of conventional Doppler and tissue Doppler echocardiography. The group of hypertensive patients included in our study was selected to have normal systolic function (EF > 50%) and early diastolic dysfunction (grade I) as assessed by the mitral inflow ratio (E/A</0.8 plus E</50 cm/sec). Furthermore, all patients included in the study were below 65 years old in order to avoid the effect of aging on diastolic function. They also had normal levels of BNP (<100 ng/ml) and E/E′ ratio < 14 indicating normal diastolic filling pressures. In this study, we demonstrate for the first time that CT-1 levels are raised in this subset of hypertensive patients with mild diastolic dysfunction, suggesting that this biomarker may have a favorable predictive significance in the early stages of hypertensive heart disease.

Previous studies have shown that HF, arterial hypertension, aortic stenosis, moderate to severe mitral regurgitation, myocardial infarction, and unstable angina may elevate CT-1 levels [1,7,8,12,17,18]. In cases of chronic HF, elevated levels of CT-1 correlate with elevated IL-6 levels, which is shown to suppress cardiac contractility and worsen endothelial function [8]. In a cohort of patients with diastolic HF, the elevated CT-1 levels were correlated with NT-proBNP and estimated left ventricular filling pressures [19]. Interestingly, CT-1 is shown to have in vitro pro-fibrotic actions [20]. López et al. have associated high levels of CT-1 with increased collagen deposition in the myocardium of hypertensive patients with HF [21]. The same authors suggested that hypertensive HF may lead to abnormal production of CT-1 by the cardiomyocytes as a response to the increasing left ventricular end-diastolic stress, stimulating the fibroblasts and causing myocardial fibrosis [21]. In fact, in our study, CT-1 levels were significantly elevated in hypertensive patients with E/E′ > 8 and <14, which indicates borderline diastolic filling pressures. This implies that CT-1 levels may respond even to mild increases in LV diastolic filling pressures and could be a very sensitive indicator. It should be noted that the ratio E/E′ was shown to independently predict primary cardiac events in a patient with controlled hypertension in a 4.2-year follow-up [22].

The potential diagnostic value of CT-1 for LV hypertrophy suggests that it can be used to monitor the onset and course of hypertensive heart disease. [18]. A number of clinical studies support this notion. In comparison to normotensive controls, hypertension patients' CT-1 levels were shown to be greater, and they were even higher in the presence of LV hypertrophy [23]. In contrast to our study, which could not confirm the association between CT-1 levels and LV hypertrophy, other studies demonstrated a cross-sectional correlation between CT-1 levels in hypertensive patients and an unreasonably high LV mass [24], along with clinically evident HF [25]. In addition, a decrease in CT-1 levels after treatment was associated with parallel regression of LV hypertrophy in treated hypertensive patients [18]. Accordingly, Ravassa et al. found that CT-1 levels were increased in asymptomatic hypertensive patients as compared to normotensives, and there was a substantial increment of CT-1 levels in patients with LV hypertrophy [26]. Since CT-1 expression is induced by hypoxia [27,28] and there was an association of CT-1 with myocardial systolic dysfunction in these patients, the authors propose that the induction of CT-1 is driven by subclinical myocardial ischemia in the context of LV hypertrophy [26]. The population included in this study is different because it was designed to include only hypertensive patients with mild diastolic dysfunction and without elevated diastolic filling pressures. In this regard, patients with LV hypertrophy and concomitant subclinical myocardial ischemia could have been excluded explaining this discrepancy.

Limitations

At the time of the study design and implementation between 2009 and 2011, tricuspid regurgitation velocities were not an important part of the diastolic assessment in our hospital. Thus, the aforementioned measurement was not included.

Taking into account that we targeted a specific group of hypertensive patients with only mild diastolic dysfunction, our sample represents only a small proportion of all hypertensive patients. Furthermore, antihypertensive treatment could be a confounding factor in the interpretation of results. Previous data, however, demonstrated that both the CT-1 levels and any association between CT-1 and LV functional measures were unaffected by the use of antihypertensive medication [26]. Lastly, even if a direct correlation was previously reported between circulating CT-1 concentration and myocardial CT-1 expression in humans [29], the contribution of potential extra-cardiac sources of circulating CT-1 in our population cannot be excluded.

## Conclusions

Our study demonstrated elevated CT-1 levels in a cohort of asymptomatic hypertensive patients, exhibiting mild diastolic dysfunction. The results of this small study provide evidence for a potential prognostic value of cardiotrophin-1 (CT-1) in monitoring the development of HF in asymptomatic hypertensive patients. As this is an ongoing research field, large clinical studies are needed in order to provide solid and evidence-based conclusions for the use of this biomarker in clinical practice.
